# Frailty trajectory among community-dwelling middle-aged and older adults in Korea: evidence from the Korean Longitudinal Study of Aging

**DOI:** 10.1186/s12877-022-03229-7

**Published:** 2022-06-25

**Authors:** Ah Ram Jang, Hae Sagong, Ju Young Yoon

**Affiliations:** 1 Center for Human-Caring Nurse Leaders for the Future By Brain Korea 21 (BK 21) four project, College of Nursing, Seoul National University, Seoul, Republic of Korea; 2 School of Nursing, Auburn University, Auburn, AL USA; 3 College of Nursing and Research Institute of Nursing Science, Seoul National University, Daehak-ro 103, Jongno-gu, 03080 Seoul, Republic of Korea

**Keywords:** Middle aged, Aged, Frailty, Trajectory

## Abstract

**Background:**

There is no study on the frailty trajectory including both middle-aged and older people, and the understanding of the long-term frailty trajectory is insufficient. This study aimed to identify the frailty trajectory, subgroups of the frailty trajectory, and the predictors that differentiate these subgroups among community-dwelling middle-aged and older adults.

**Methods:**

The participants were 9,775 individuals aged 45 years and older who participated in the Korean Longitudinal Study of Aging (2006–2018). Frailty was measured using a frailty instrument comprising three items: grip strength weakness, exhaustion, and social isolation. Latent growth curve modeling and latent class growth modeling were performed to identify the frailty trajectory and latent classes of the trajectory. Multinomial logistic regression was used to confirm the predictors that classified the latent classes.

**Results:**

Over 12 years, the slope of the frailty trajectory among the participants showed a gradual increase. In addition, there was a difference in the latent class of frailty trajectories among middle-aged and older adults. The middle-aged participants were divided into two groups: maintaining robustness and changing from pre-frailty to robustness. The older adults were divided into three groups: maintaining robustness, maintaining pre-frailty, and changing from the frailty to pre-frailty group. Regular exercise, cognitive dysfunction, and social participation were significant predictors that differentiated each latent class in both middle-aged and older adults; additionally, current smoking and the number of chronic diseases were significant predictors in middle-aged people.

**Conclusions:**

Various subgroups within the frailty trajectory existed among community-dwelling middle-aged and older adults. To reduce frailty, it is necessary to intervene with modifiable factors appropriate for each age group.

**Supplementary Information:**

The online version contains supplementary material available at 10.1186/s12877-022-03229-7.

## Background

Frailty is a dynamic condition that affects individuals experiencing loss in one or more of the physical, psychological, or social domains [1]. Frailty is influenced by multiple variables, and increases the risk of negative health outcomes including mortality, falls, hospitalization, and disability in performing activities of daily living [1–3].

Frailty is not a static state but a dynamic state that can worsen or improve over time [4]. Several studies have investigated factors related to frailty changes. Frailty progression is influenced by various factors, particularly, social demographics, brain pathology, and physical comorbidities [5]. Specifically, demographic factors including age, sex, and education [6] and diseases such as diabetes [7] and osteoporotic fractures [4] affect frailty trajectory. In addition, vigorous physical activity significantly reduces frailty progression [8], and cognitive decline [9] was also found to influence the frailty trajectory.

Furthermore, there is heterogeneity between individuals in the frailty trajectory and there are subgroups with various change patterns [10]. In an 18-year longitudinal study of 1,362 Mexican-Americans aged 65 years and older, the frailty trajectory was found to have three subgroups: non-frail, moderate progressive, and progressive high [10]. According to a study analyzing the relationship between frailty trajectory and mortality, the rapid rising and moderately increasing frailty group increased the mortality rate by 180% and 65%, respectively, compared to the stable frailty group [11]. Therefore, it is urgent to identify and intervene in people at high risk for frailty progression to prevent negative health consequences.

However, to date, studies that identify subgroups of frailty trajectories longitudinally and predictors that differentiate trajectory patterns are very limited. In addition, previous studies on frailty trajectories have been conducted only on the elderly, and studies on middle-aged individuals have been neglected. A recent study reported that the prevalence of pre-frailty and frailty among people aged 40–49 years was 45% and 1.4%, respectively, similar to those of people aged 70–75 years, and interventions should be initiated at the age of 40 to prevent frailty [12]. However, there is still no study on the frailty trajectory including both middle-aged and older people, and the understanding of the long-term frailty trajectory is insufficient. Thus, this study aimed to identify the frailty trajectory, subgroups of the frailty trajectory, and predictors that differentiate these subgroups among community-dwelling adults aged 45 years or older using the Korean Longitudinal Study of Aging (KLoSA).

## Methods

### Data and participants

The KLoSA is a longitudinal panel survey of community-dwelling older adults aged ≥ 45 years in South Korea. The first survey was conducted in 2006, and then performed every two years, of which the seventh survey was completed in 2018. The questionnaire items were broadly structured, such as demographics, family characteristics, physical and mental health, and employment. To represent the Korean population, households stratified by region and residential type were selected using simple random sampling. The interview was conducted using a computer-assisted personal interviewing technique. In this study, data of all waves from the first (2006) to the seventh survey (2018) were used. The total number of participants in 2006 was 10,254, of which 9,775 who participated in the survey twice or more were included as the final participants in this study.

KLoSA is public; anonymized data can be accessed by anyone who requests the data from an address (https://survey.keis.or.kr). The study procedures were reviewed and approved by the Institutional Review Board (IRB) of Seoul National University. We received a waiver of informed consent, and all research procedures were performed after IRB approval (IRB No. E2105/002–006). This study was conducted in accordance with the principles of the Declaration of Helsinki.

### Measures

Frailty was measured using the frailty instrument (FI), which defines frailty broadly in terms of physical phenotype and psychological and social aspects. FI consisted of three items assessing weakness in grip strength, exhaustion, and social isolation. For grip strength weakness, 1 point was given to less than 15 kg for women and less than 24 kg for men. For exhaustion, 1 point was given if the self-reported response was more than 3 days in one or more of the following two questions: “I feel difficult about everything” and “I cannot do anything at all” in the past week. Social isolation was given 1 point if the respondents answered that they did not participate in any of the following groups: social, religious, cultural, sports, civic, political, volunteer, and learning groups. The total range of the frailty score ranged from 0 to 3 and was categorized as follows: robust (0), pre-frail (≥ 1), and frail (≥ 2) [13]. The FI has been validated in the Korean elderly and shows high predictive validity, discrimination, and calibration ability for adverse health outcomes such as disability, institutionalization, and mortality [13, 14].

General characteristics included age (years), education level (less than elementary school or more than middle school), marital status (married or single/widowed/divorced/unmarried), and area of residence (urban or rural). Lifestyle-related factors such as smoking (currently smoking or never/used to but not now), drinking (currently drinking or never/used to but not now), and regular physical activity at least once a week (yes or no) were also assessed. Regarding chronic diseases, participants were asked about the number of chronic diseases diagnosed by their physicians. Cognitive function was measured using the Korean version of the Mini-Mental State Examination (K-MMSE) validated in the Korean population [15]. The K-MMSE scores ranged 0–30, with a score of less than 24 regarded as cognitive dysfunction. Social contact refers to the number of times participants meet with their close acquaintances. Participants answered on a 10-point Likert scale from 1 (no one to get along with) to 10 points (meeting almost every day), with higher scores indicating active social contact.

### Statistical analysis

Baseline characteristics, according to the sex of the middle-aged and older adults, were compared using a χ2-test and an independent t-test for categorical and continuous variables, respectively.

Latent growth curve modeling (LGCM), which can quantify individual change over time, was performed to identify the frailty trajectory from 2006 to 2018. The mean and variance of the intercept, slope, and quadratic term of the frailty trajectory were estimated by applying an unconditional model analysis without covariates. The goodness of fit was confirmed using chi-square values, comparative fit index (CFI), Tucker–Lewis index (TLI), root mean square error of approximation (RMSEA), and standardized root mean square residual (SRMR).

Latent class growth modeling (LCGM), which combines the latent growth model and the latent class model, was used to confirm the latent class type for the frailty trajectory. To determine the number of latent classes of middle-aged and older adults, various model fit indices were used. The Akaike information criterion (AIC), Bayesian information criterion (BIC), adjusted BIC, negative log likelihood, entropy, Lo‐Mendell‐Rubin likelihood ratio test (LMR), and proportions for the latent classes were assessed, and the model with the best fit indices was selected. After identifying the latent classes with different frailty trajectories, multinomial logistic regression was performed to confirm the predictors that classified the classes. Full-information maximum likelihood (FIML) was used to handle missing data.

Descriptive and multinomial logistic regression analyses were performed using SPSS (version 26.0; IBM, Armonk, NY, USA). LGCM and LCGM were performed using Mplus version 8.6.

## Results

The baseline characteristics of the participants are presented in Table 1. In the middle-aged, the mean age was 53.96 ± 5.86 years, and males represented 44.6% of the group. In older adults, the mean age was 72.45 ± 5.88 years, and males accounted for 42.9% of the group. Using the LGCM, we analyzed the change in frailty over 12 years (Table 2). The slope of the frailty trajectory among participants showed a gradual increase. The mean variance of the intercept, slope, and quadratic terms were all significant, indicating significant individual differences in the frailty trajectory. Therefore, it was determined that there would be several latent classes showing a heterogeneous change pattern according to the frailty trajectory, and LCGM was applied to estimate these latent classes.

**Table 1:** Baseline characteristics of participants (*n* = 9775)

Variables	Total (*n* = 9775)	Middle-aged (*n* = 5999)			Older adults (*n* = 3776)		
		Male (*n* = 2676)	Female (*n* = 3323)	*P*-value	Male (*n* = 1620)	Female (*n* = 2156)	*P*-value
**Age (years)**	61.10 ± 10.75	54.09 ± 5.79	53.86 ± 5.91	0.125	71.92 ± 5.48	72.85 ± 6.14	< 0.001
**Education**							
≥ Middle school	5312 (54.3)	2184 (81.7)	2047 (61.7)	< 0.001	779 (48.1)	302 (14.0)	< 0.001
< Middle school	4455 (45.6)	490 (18.3)	1273 (38.3)		840 (51.9)	1852 (86.0)	
**Marital status**							
Married	7722 (79.0)	2490 (93.0)	521 (15.7)	< 0.001	1463 (90.3)	967 (44.9)	< 0.001
Single/divorced/widowed	2053 (21.0)	186 (7.0)	2802 (84.3)		157 (9.7)	1189 (55.1)	
**Area of residence**							
Urban	7574 (77.5)	2189 (81.8)	2699 (81.2)	0.566	1131 (69.8)	1555 (72.1)	0.121
Rural	2201 (22.5)	487 (18.2)	624 (18.8)		489 (30.2)	601 (27.9)	
**Smoking**							
Currently non-smoker	7859 (80.4)	1441 (53.8)	3234 (97.3)	< 0.001	1112 (68.7)	2072 (96.1)	< 0.001
Current smoker	1915 (19.6)	1235 (46.2)	89 (2.7)		507 (31.3)	84 (3.9)	
**Drinking**							
No	5960 (61.0)	772 (28.8)	2530 (76.1)	< 0.001	758 (46.8)	1900 (88.1)	< 0.001
Yes	3815 (39.0)	1904 (71.2)	793 (23.9)		862 (53.2)	256 (11.9)	
**Regular physical activity**							
Yes	3858 (39.5)	1200 (44.8)	1399 (42.1)	0.033	675 (41.7)	584 (27.1)	< 0.001
No	5917 (60.5)	1476 (55.2)	1924 (57.9)		945 (58.3)	1572 (72.9)	
**Number of chronic diseases (0–10)**	0.73 ± 0.94	0.47 ± 0.79	0.54 ± 0.83	0.001	0.93 ± 0.98	1.16 ± 1.05	< 0.001
**Cognitive function**							
Normal	7487 (76.6)	2468 (93.5)	2863 (87.1)	< 0.001	1200 (74.5)	956 (44.9)	< 0.001
Cognitive dysfunction	2177 (22.3)	171 (6.5)	425 (12.9)		410 (25.5)	1171 (55.1)	
**Social contact (1–10)**	7.49 ± 2.85	7.29 ± 2.71	7.62 ± 2.75	< 0.001	7.41 ± 3.01	7.60 ± 3.02	0.052
**Frailty instrument scores (0–3)**							
Survey 1: 2006 (*n* = 9386)	0.55 ± 0.77	0.31 ± 0.56	0.39 ± 0.62	< 0.001	0.73 ± 0.86	0.99 ± 0.90	< 0.001
Survey 2: 2008 (*n* = 7717)	0.54 ± 0.78	0.26 ± 0.53	0.37 ± 0.63	< 0.001	0.76 ± 0.84	1.03 ± 0.95	< 0.001
Survey 3: 2010 (*n* = 6936)	0.60 ± 0.80	0.35 ± 0.62	0.42 ± 0.67	< 0.001	0.85 ± 0.86	1.09 ± 0.91	< 0.001
Survey 4: 2012 (*n* = 6411)	0.56 ± 0.76	0.34 ± 0.58	0.39 ± 0.64	0.003	0.81 ± 0.84	1.02 ± 0.90	< 0.001
Survey 5: 2014 (*n* = 5846)	0.54 ± 0.76	0.34 ± 0.60	0.39 ± 0.66	0.007	0.80 ± 0.83	1.03 ± 0.92	< 0.001
Survey 6: 2016 (*n* = 5436)	0.54 ± 0.77	0.34 ± 0.60	0.43 ± 0.68	< 0.001	0.85 ± 0.91	0.98 ± 0.92	0.007
Survey 7: 2018 (*n* = 4954)	0.52 ± 0.75	0.33 ± 0.58	0.38 ± 0.64	0.008	0.96 ± 0.87	0.99 ± 0.93	0.598
**Frailty instrument components**							
**A. Weakness of grip strength**							
No	8012 (82.0)	2515 (96.7)	3003 (93.1)	< 0.001	1190 (75.5)	1304 (64.7)	< 0.001
Yes	1407 (14.4)	87 (3.3)	222 (6.9)		386 (24.5)	712 (35.3)	
**B. Exhaustion**							
No	8488 (86.8)	2482 (93.1)	3008 (91.0)	0.751	1369 (84.7)	1629 (75.9)	< 0.001
Yes	1245 (12.7)	184 (6.9)	297 (9.0)		248 (15.3)	516 (24.1)	
**C. Social isolation**							
No	7023 (71.8)	2103 (78.6)	2552 (76.8)	0.098	1074 (66.3)	1294 (60.0)	< 0.001
Yes	2752 (28.2)	573 (21.4)	771 (23.2)		546 (33.7)	862 (40.0)	
**Combinations of frailty instrument components**							
**A. Weakness of grip strength + exhaustion**							
No	9258 (94.7)	2636 (99.1)	3241 (98.2)	0.007	1492 (92.8)	1889 (89.6)	0.001
Yes	417 (4.3)	25 (0.9)	58 (1.8)		115 (7.2)	219 (10.4)	
**B. Weakness of grip strength + social isolation**							
No	9015 (92.2)	2618 (98.9)	3217 (97.7)	< 0.001	1430 (89.0)	1750 (83.5)	< 0.001
Yes	628 (6.4)	29 (1.1)	76 (2.3)		177 (11.0)	346 (16.5)	
**C. Exhaustion + social isolation**							
No	9160 (93.7)	2583 (96.7)	3202 (96.5)	0.714	1483 (91.5)	1892 (88.0)	< 0.001
Yes	599 (6.1)	88 (3.3)	115 (3.5)		137 (8.5)	259 (12.0)	

Data are shown as the mean ± SD or n (%). Middle-aged, participants aged < 65 years at baseline; Older adults, participants aged ≥ 65 years at baseline

**Table 2:** Results of the frailty trajectory among participants using latent growth curve modeling (*n* = 9775)

			Mean (SE)	Variance (SE)	Model fit				
					χ^2^ (*df*)	CFI	TLI	RMSEA	SRMR
Middle-aged	Male	Intercept	0.291*(0.010)	0.136*(0.011)	174.979*(19)	0.939	0.932	0.055	0.041
		Slope	0.029*(0.008)	0.034*(0.005)					
		Quadratic	-0.003*(0.001)	0.001*(0.000)					
	Female	Intercept	0.383*(0.010)	0.166*(0.012)	132.501*(19)	0.970	0.967	0.042	0.029
		Slope	0.020*(0.007)	0.035*(0.005)					
		Quadratic	-0.002*(0.001)	0.001*(0.000)					
Older adults	Male	Intercept	0.740*(0.021)	0.409*(0.035)	69.537*(19)	0.965	0.962	0.041	0.039
		Slope	0.057*(0.016)	0.094*(0.017)					
		Quadratic	0.000(0.003)	0.002*(0.000)					
	Female	Intercept	0.998*(0.019)	0.384*(0.034)	47.571*(19)	0.987	0.985	0.026	0.033
		Slope	0.070*(0.014)	0.073*(0.016)					
		Quadratic	-0.009*(0.002)	0.002*(0.000)					

Middle-aged, participants aged < 65 years at baseline; Older adults, participants aged ≥ 65 years at baseline. *SE* standard error, *CFI* comparative fit index, *TLI* Tucker–Lewis index, *RMSEA* root mean square error of approximation, *SRMR* standardized root mean square residual. *p < .001

LCGM was performed to determine the number of latent subgroups according to the frailty trajectories. In middle-aged individuals, the LMR *P* value of the three-class model was not significant, and the number of class 3 samples was too small to make a conceptual sense [16] (Supplementary Table 3). Therefore, the two-class model was selected as a suitable model. In older adults, the number of samples in class 4 in the four-class model was small (Supplementary Table 4). Additionally, the BIC and adjusted BIC values of the four-class model increased compared to that of the three-class model in the female group, so the three-class model was selected as the optimal model.

**Fig. 1:**
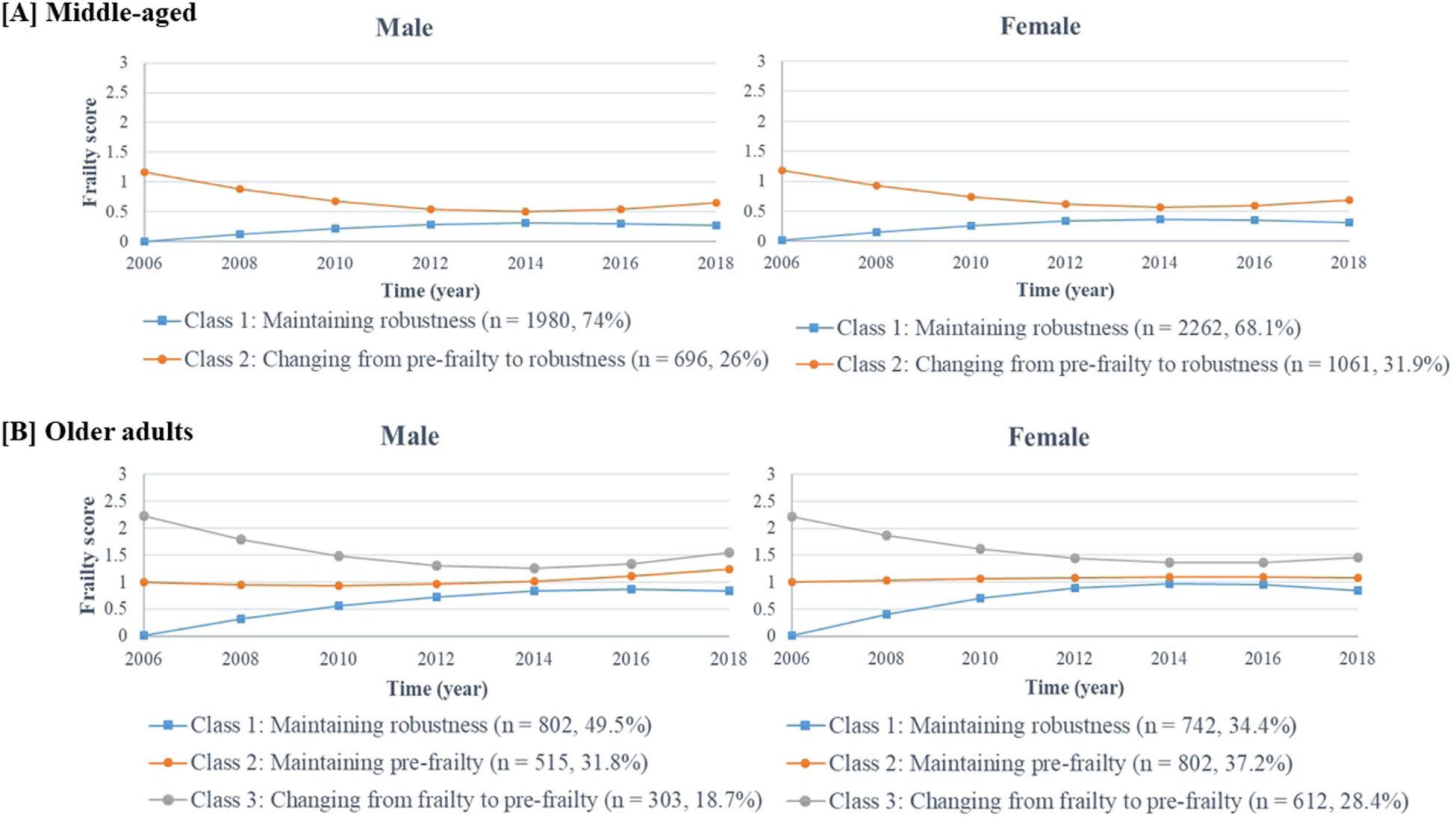
Growth trajectories of frailty by each latent class of middle-aged [A] and older adults [B] according to sex. *Note.* Middle-aged, participants aged < 65 years at baseline; Older adults, participants aged ≥ 65 years at baseline. A higher frailty score indicates a severe frailty status. **A.** male Class 1: intercept = 0.003*, slope = 0.141*, quadratic = -0.016*; Class 2: intercept = 1.163*, slope = -0.324*, quadratic = 0.040*. **A.** female Class 1: intercept = 0.012*, slope = 0.164*, quadratic = -0.019*; Class 2: intercept = 1.185*, slope = -0.294*, quadratic = 0.035*. **B.** male Class 1: intercept = 0.004*, slope = 0.342*, quadratic = -0.034*; Class 2: intercept = 0.999*, slope = -0.068*, quadratic = 0.018*; Class 3: intercept = 2.225*, slope = -0.503*, quadratic = 0.065*. **B.** female Class 1: intercept = 0.005*, slope = 0.451*, quadratic = -0.052*; Class 2: intercept = 1.000*, slope = 0.044*, quadratic = -0.005*; Class 3: intercept = 2.218*, slope = -0.391*, quadratic = 0.044 *. *p < .05

The patterns of the frailty trajectories in each latent class are presented in Fig. 1. In middle-aged individuals, both males and females were divided into two groups: maintaining robustness and changing from pre-frailty to robustness. In older adults, both sexes were divided into three groups: maintaining robustness, maintaining pre-frailty, and changing from frailty to pre-frailty.

Factors predicting membership in latent classes were identified using multinomial logistic regression analysis. In both middle-aged males and females (Table 3), those who had lower than middle school education, were single/divorced/widowed, were currently smoking, did not exercise regularly, were afflicted with several chronic diseases, had cognitive dysfunction, and had low social contact were more likely to belong to the changing from pre-frailty to robustness group. In both older men and women (Table 4), participants who were older, did not exercise regularly, had cognitive dysfunction, had low social contact, were more likely to belong to the changing from frailty to pre-frailty group and less likely to belong to the maintaining robustness group.

**Table 3:** Multinomial logistic regression analysis predicting membership of latent classes in middle-aged (*n* = 5999)

Variables	Male	Female
	Class 2: Changing from pre-frailty to robustness (*n* = 696)	Class 2: Changing from pre-frailty to robustness (*n* = 1061)
	OR (95% CI)	OR (95% CI)
**Age (years)**	1.022 (1.003–1.040)	1.002 (0.986–1.018)
**Education**		
≥ Middle school	1	1
< Middle school	1.748 (1.362–2.243)	2.041 (1.690–2.465)
**Marital status**		
Married	1	1
Single/divorced/widowed	3.673 (2.607–5.173)	1.517 (1.225–1.877)
**Area of residence**		
Urban	1	1
Rural	1.141 (0.884–1.473)	1.249 (1.017–1.534)
**Smoking**		
Currently non-smoker	1	1
Current smoker	1.297 (1.057–1.591)	2.751 (1.679–4.506)
**Drinking**		
No	1	1
Yes	0.891 (0.716–1.109)	1.080 (0.890–1.311)
**Regular physical activity**		
Yes	1	1
No	1.654 (1.347–2.031)	1.435 (1.210–1.701)
**Number of chronic diseases (0–10)**	1.227 (1.086–1.386)	1.255 (1.136–1.387)
**Cognitive function**		
Normal	1	1
Cognitive dysfunction	1.659 (1.152–2.391)	1.459 (1.151–1.849)
**Social contact (1–10)**	0.757 (0.731–0.785)	0.801 (0.778–0.825)

The reference group is class 1(maintaining robustness) of each age group. *OR* odds ratio, *CI* confidence interval

**Table 4:** Multinomial logistic regression analysis predicting membership of latent classes in older adults (*n* = 3776)

Variables	Male		Female	
	Class 1: Maintaining robustness (*n* = 802)	Class 3: Changing from frailty to pre-frailty (*n* = 303)	Class 1: Maintaining robustness (*n* = 742)	Class 3: Changing from frailty to pre-frailty (*n* = 612)
	OR (95% CI)		OR (95% CI)	
**Age (years)**	0.959 (0.937–0.982)	1.050 (1.022–1.078)	0.971 (0.951–0.991)	1.037 (1.018–1.057)
**Education**				
≥ Middle school	1	1	1	1
< Middle school	0.576 (0.447–0.742)	1.532 (1.080–2.173)	0.730 (0.538–0.990)	1.479 (0.981–2.229)
**Marital status**				
Married	1	1	1	1
Single/divorced/widowed	0.770 (0.505–1.172)	1.976 (1.258–3.102)	0.772 (0.616–0.966)	1.178 (0.925–1.500)
**Area of residence**				
Urban	1	1	1	1
Rural	1.104 (0.835–1.459)	1.368 (0.967–1.937)	0.814 (0.637–1.041)	1.016 (0.791–1.305)
**Smoking**				
Currently non-smoker	1	1	1	1
Current smoker	0.713 (0.552–0.922)	0.990 (0.710–1.380)	0.537 (0.284–1.015)	1.178 (0.710–1.954)
**Drinking**				
No	1	1	1	1
Yes	1.391 (1.096–1.765)	0.854 (0.625–1.166)	1.104 (0.801–1.523)	0.849 (0.597–1.207)
**Regular physical activity**				
Yes	1	1	1	1
No	0.760 (0.590–0.980)	1.917 (1.339–2.746)	0.686 (0.540–0.872)	1.361 (1.024–1.807)
**Number of chronic diseases (0–10)**	0.966 (0.854–1.094)	1.313 (1.128–1.528)	0.913 (0.825–1.010)	1.097 (0.990–1.215)
**Cognitive function**				
Normal	1	1	1	1
Cognitive dysfunction	0.653 (0.487–0.876)	2.002 (1.445–2.773)	0.540 (0.429–0.679)	1.379 (1.075–1.770)
**Social contact (1–10)**	1.183 (1.134–1.235)	0.909 (0.868–0.952)	1.168 (1.119–1.219)	0.906 (0.875–0.938)

The reference group is class 2 (maintaining pre-frailty) of each age group. *OR* odds ratio, *CI* confidence interval

## Discussion

In this study, the frailty trajectory among community-dwelling middle-aged and older adults was analyzed longitudinally using KLoSA data from 2006 to 2018. Specifically, the frailty trajectory was confirmed using LGCM, and it was found that frailty became more severe over time in all age groups. In addition, we found different latent classes in frailty trajectories for each age group using the LCGM. In middle-aged individuals, a total of two trajectories were found, maintaining robustness and changing from pre-frailty to robustness. In the change from pre-frailty to robustness subgroup, the participants showed initial pre-frailty but then improved to robustness. These results are consistent with previous studies showing that younger people are more likely to improve from frailty [17]. In addition, the middle-aged group in this study had a lower frailty score than that of the older adults’ group, and there was no subgroup within the trajectory corresponding to frailty status. This indicates that the incidence of frailty in middle age is low. However, another possible explanation is that frailty is an age-related geriatric syndrome [18], so the trajectory of frailty due to aging may not be well revealed in middle-aged individuals.

The older adults were divided into three groups. Unlike middle-aged individuals, most of them were initially frail or pre-frail. In addition, there was no improvement from pre-frailty or frailty to the robustness group, only maintenance or slight improvement. While frailty is a dynamic condition [4], it worsens with age [17], suggesting that it is difficult to improve into a robust group, especially for the older adults. In a previous 18-year longitudinal study of the Mexican American elderly, the frailty trajectory was classified into three categories: non-frail, moderate progressive, and progressive high [10]. Analysis of these categories revealed the presence of three subgroups, similar to the ones found in our study. However, their trajectory patterns differed from those in our study since there were groups that deteriorated over time. As there are few longitudinal studies to identify subgroups within the frailty trajectory, the exact mechanism for the difference in these results is not well known, but it may be due to attrition. As such, it is necessary to acquire more evidence through longitudinal studies in the future.

Multinomial logistic regression analysis was used to identify predictors that differentiated latent classes. Age was a predictor that differentiated the latent classes of middle-aged males and older adults. A previous systematic review showed that age is frequently associated with frailty levels and changes in frailty status [5]. In a 4-year longitudinal study, an increase in age was associated with the occurrence of frailty [19], supporting the results of this study. Educational level was a significant predictor in both middle-aged and older adults. These results correspond well with a 10-year follow-up study that revealed that low educational levels increased the likelihood of frailty [20]. Also, marital status was a significant predictor that differentiated each class in middle-aged and older adults, showing the similarity to those found in the earlier study [21]. As a result of a meta-analysis, unmarried individuals were twice as likely to be frail than married individuals [21]. The exact mechanism of why unmarried people are getting frailer than married people is unknown, but it has been reported that stress from widowhood, divorce, or separation may increase frailty [21]. Divorce or being unmarried may also lead to unhealthy behaviors, such as heavy drinking or smoking [22], which may be linked to frailty.

Current smoking had a significant effect on the difference in frailty trajectories of older adult males and middle-aged adults, similar to the results of a 4-year longitudinal study [23]. Smoking causes various diseases such as cancer, heart attack, coronary heart disease, and lung diseases [24], which can affect frailty. Interestingly, in older males, current drinkers were more likely to belong to the maintaining robustness group, similar to the result of a meta-analysis that heavy drinking lowered the incidence of frailty [25]. In contrast, these findings differ from those of a 3-year longitudinal study that heavy drinking was not associated with decreased risk of frailty [26]. The results in this study may be due to unadjusted effect measures, residual confounding, sick quitter effect, or survival bias; therefore, caution is needed in the interpretation [25]. Lack of regular physical activity is a predictor influencing the frailty trajectory in both middle-aged and older adults, consistent with a previous study [8].

The number of chronic diseases also had a significant effect on classification into frailty groups in middle-aged and older adult males. The two concepts of chronic disease and frailty are related to each other and chronic diseases can contribute to the occurrence of frailty [27]. Cognitive dysfunction was also found to be a strong risk factor that differentiated each group, consistent with the results of previous literature [9, 28]. Frailty and cognitive decline share risk factors including chronic disease, poor cardiovascular health, inflammation, or hormonal dysregulation [28]. Also, behavioral changes due to cognitive decline can lead to frailty through reduced physical activity and nutritional deficiencies [28]. Furthermore, social contact was a significant predictor that differentiated between each group in both middle-aged and older adults, which is in agreement with a previous study reporting that less frequent contact and high levels of loneliness increased the likelihood of frailty [29].

This study has some limitations. First, since the independent variable was used from the baseline, the change in the independent variable during the study period could not be considered. Second, caution is needed when interpreting the results as missing data occur in longitudinal studies. Those excluded from the study were older and had higher rates of chronic diseases and cognitive dysfunction (Supplementary Tables 1 and 2), which may have resulted in an underestimation of frailty. Third, since there are no frailty measuring tools made exclusively for middle-aged people, the FI, which was developed for the older adults, was used to identify frailty among the middle-aged. This may have underestimated the frailty of the middle-aged, suggesting the need to develop a frailty tool targeting this population in the future. Lastly, we only used the FI for the measurement of frailty because the available variables in the KLoSA were limited. Future studies are needed to compare the difference in the frailty trajectory using widely used instruments such as the Cardiovascular Health Study or Study of Osteoporotic Fractures Index. Nevertheless, to the best of our knowledge, this is the first study to identify various change patterns in frailty trajectories and predictors causing such different patterns in middle-aged and older adults. The present study contributes to the understanding of the long-term trajectory of frailty and provides new insights to prevent the progression of frailty. In addition, this study can be generalized to the community-dwelling population because we used Korean big data with low selection bias and high representativeness of the population.

## Conclusions

In conclusion, various subgroups within the frailty trajectory existed in the community-dwelling middle-aged and older adults. The middle-aged and older adults were divided into two and three groups, respectively. Most of the middle-aged people were in the maintaining robustness group, and those who were pre-frail at the beginning also showed a tendency to return to the robustness group as time passed. On the other hand, most older adults were initially in a state of pre-frailty or frailty, and there was no improvement to the robustness group over time; therefore, preventing or delaying the onset of frailty is necessary for the older adults because it is likely that the condition will continue once it commences.

In addition, to maintain a robust state, interventions focusing on modifiable factors such as smoking cessation, regular exercise, prevention of chronic diseases, cognitive function improvement, and social participation enhancement are necessary for middle-aged individuals. For older adults, interventions targeting regular exercise, cognitive function improvement, and social participation enhancement are necessary to maintain a robust state and prevent frailty.

## Supplementary Information

**Additional file 1: Supplementary Table 1. **Baseline characteristics of included and excluded middle-aged, mean ± SD or n (%). **Supplementary Table 2. **Baseline characteristics of included and excluded older adults, mean ± SD or n (%).**Supplementary Table 3. **Model fit for latent class growth modeling in middle-aged (*n* = 5999). **Supplementary Table 4. **Model fit for latent class growth modeling in older adults (*n* = 3776).

## Data Availability

The data cannot be shared publicly because there was no such approval in the study protocol. The datasets used and analyzed during the study are available from the corresponding author on reasonable request.

## Abbreviations

KLoSA: Korean Longitudinal Study of Aging
IRB: Institutional Review Board
FI: Frailty instrument
K-MMSE: Korean version of the Mini-Mental State Examination
LGCM: Latent growth curve modeling
CFI: Comparative fit index
TLI: Tucker–Lewis index
RMSEA: Root mean square error of approximation
SRMR: Standardized root mean square residual
LCGM: Latent class growth modeling
AIC: Akaike information criterion
BIC: Bayesian information criterion
LMR: Lo‐Mendell‐Rubin likelihood ratio test
FIML: Full-information maximum likelihood
