# Supplementary material for: Frailty trajectory among community-dwelling middle-aged and older adults in Korea: evidence from the Korean Longitudinal Study of Aging
Source: BMC Geriatr. 2022 Jun 25;22:524. doi: 10.1186/s12877-022-03229-7 (PMC9233334; doi:10.1186/s12877-022-03229-7)
Supplement: Supplementary file 1 — Additional file 1: Supplementary Table 1. Baseline characteristics of included and excluded middle-aged, mean ± SD or n (%). Supplementary Table 2. Baseline characteristics of included and excluded older adults, mean ± SD or n (%).Supplementary Table 3. Model fit for latent class growth modeling in middle-aged (n = 5999). Supplementary Table 4. Model fit for latent class growth modeling in older adults (n = 3776). [file 12877_2022_3229_MOESM1_ESM.docx]

**Supplementary Table 1.** Baseline characteristics of included and excluded middle-aged, mean ± SD or n (%)

| Variables | Included  (*n* = 5999) | Excluded  (*n* = 91) | *P* |
| --- | --- | --- | --- |
| **Age (years)** | 53.96 ± 5.86 | 55.59 ± 6.41 | 0.018 |
| **Sex** |  |  |  |
| Male | 2676 (44.6) | 46 (50.5) | 0.258 |
| Female | 3323 (55.4) | 45 (49.5) |  |
| **Education** |  |  |  |
| ≥ Middle school | 4231 (70.6) | 56 (61.5) | 0.060 |
| < Middle school | 1763 (29.4) | 35 (38.5) |  |
| **Marital status** |  |  |  |
| Married | 5292 (88.2) | 70 (76.9) | 0.001 |
| Single/divorced/widowed | 707 (11.8) | 21 (23.1) |  |
| **Area of residence** |  |  |  |
| Urban | 4888 (81.5) | 81 (89.0) | 0.066 |
| Rural | 1111 (18.5) | 10 (11.0) |  |
| **Smoking** |  |  |  |
| Currently non-smoker | 4675 (77.9) | 72 (79.1) | 0.786 |
| Current smoker | 1324 (22.1) | 19 (20.9) |  |
| **Drinking** |  |  |  |
| No | 3302 (55.0) | 69 (75.8) | <0.001 |
| Yes | 2697 (45.0) | 22 (24.2) |  |
| **Regular physical activity** |  |  |  |
| Yes | 2599 (43.3) | 23 (25.3) | 0.001 |
| No | 3400 (56.7) | 68 (74.7) |  |
| **Number of chronic diseases (0–10)** | 0.51 ± 0.81 | 1.11 ± 1.06 | <0.001 |
| **Cognitive function** |  |  |  |
| Normal | 5331 (89.9) | 39 (55.7) | <0.001 |
| Cognitive dysfunction | 596 (10.1) | 31 (44.3) |  |
| **Social contact (1–10)** | 7.47 ± 2.74 | 5.27 ± 3.60 | <0.001 |
| **Frailty instrument scores (0–3)** | 0.32 ± 0.59 | 1.00 ± 1.03 | 0.018 |
| *Note.* SD, standard deviation. | |  |  |

**Supplementary Table 2.** Baseline characteristics of included and excluded older adults, mean ± SD or n (%)

| Variables | Included  (*n* = 3776) | Excluded  (*n* = 388) | *P* |
| --- | --- | --- | --- |
| **Age (years)** | 72.45 ± 5.88 | 78.31 ± 7.69 | <0.001 |
| **Sex** |  |  |  |
| Male | 1620 (42.9) | 121 (31.2) | <0.001 |
| Female | 2156 (57.1) | 267 (68.8) |  |
| **Education** |  |  |  |
| ≥ Middle school | 1081 (28.7) | 54 (13.9) | <0.001 |
| < Middle school | 2692 (71.3) | 334 (86.1) |  |
| **Marital status** |  |  |  |
| Married | 2430 (64.4) | 179 (46.1) | <0.001 |
| Single/divorced/widowed | 1346 (35.6) | 209 (53.9) |  |
| **Area of residence** |  |  |  |
| Urban | 2686 (71.1) | 275 (70.9) | 0.915 |
| Rural | 1090 (28.9) | 113 (29.1) |  |
| **Smoking** |  |  |  |
| Currently non-smoker | 3184 (84.3) | 345 (88.9) | 0.017 |
| Current smoker | 591 (15.7) | 43 (11.1) |  |
| **Drinking** |  |  |  |
| No | 2658 (70.4) | 336 (86.6) | <0.001 |
| Yes | 1118 (29.6) | 52 (13.4) |  |
| **Regular physical activity** |  |  |  |
| Yes | 1259 (33.3) | 51 (13.1) | <0.001 |
| No | 2517 (66.7) | 337 (86.9) |  |
| **Number of chronic diseases (0–10)** | 1.06 ± 1.03 | 1.35 ± 1.13 | <0.001 |
| **Cognitive function** |  |  |  |
| Normal | 2156 (57.7) | 42 (13.7) | <0.001 |
| Cognitive dysfunction | 1581 (42.3) | 265 (86.3) |  |
| **Social contact (1–10)** | 7.52 ± 3.02 | 5.78 ± 3.53 | <0.001 |
| **Frailty instrument scores (0–3)** | 0.91 ± 0.91 | 1.71 ± 0.97 | <0.001 |
| *Note.* SD, standard deviation. | |  |  |

**Supplementary Table 3.** Model fit for latent class growth modeling in middle-aged (*n* = 5999)

|  | Model fit indices | Two-class model | Three-class model |
| --- | --- | --- | --- |
| Male | AIC | 19932.569 | 14048.593 |
|  | BIC | 20050.410 | 14190.003 |
|  | Adjusted BIC | 19986.864 | 14113.747 |
|  | Entropy | 0.983 | 0.986 |
|  | Negative LL | 9946.284 | 7000.296 |
|  | LMR *P* value | .0170 | .505 |
|  | Class counts | Class 1: 1980 (74.0%) | Class 1: 1981 (74.0%) |
|  |  | Class 2: 696 (26.0%) | Class 2: 577 (21.6%) |
|  |  |  | Class 3: 118 (4.4%) |
| Female | AIC | 29436.551 | 23479.520 |
|  | BIC | 29558.723 | 23626.127 |
|  | Adjusted BIC | 29495.174 | 23549.869 |
|  | Entropy | 0.963 | 0.981 |
|  | Negative LL | 14698.275 | 11715.76 |
|  | LMR *P* value | .043 | .088 |
|  | Class counts | Class 1: 2262 (68.1%) | Class 1: 2266 (68.2%) |
|  |  | Class 2: 1061(31.9%) | Class 2: 868 (26.1%) |
|  |  |  | Class 3: 189 (5.7%) |
| Note*.* AIC, Akaike information criterion; BIC, Bayesian information criterion; LL, log likelihood; LMR, Lo‐Mendell‐Rubin likelihood ratio test. | | | |

**Supplementary Table 4.** Model fit for latent class growth modeling in older adults (*n* = 3776)

|  | Model fit indices | Two-class model | Three-class model | Four-class model |
| --- | --- | --- | --- | --- |
| Male | AIC | 15544.344 | 14298.695 | 14260.762 |
|  | BIC | 15652.147 | 14428.059 | 14411.687 |
|  | Adjusted BIC | 15588.611 | 14351.815 | 14322.736 |
|  | Entropy | 0.859 | 0.979 | 0.906 |
|  | Negative LL | 7752.172 | 7125.347 | 7102.381 |
|  | LMR *P* value | .000 | .002 | .000 |
|  | Class counts | Class 1: 1319 (81.4%) | Class 1: 802 (49.5%) | Class 1: 802 (49.5%) |
|  |  | Class 2: 301 (18.6%) | Class 2: 515 (31.8%) | Class 2: 425 (26.2%) |
|  |  |  | Class 3: 303 (18.7%) | Class 3: 304 (18.8%) |
|  |  |  |  | Class 4: 89 (5.5%) |
| Female | AIC | 21022.810 | 20102.617 | 20100.957 |
|  | BIC | 21136.33 | 20238.841 | 20259.886 |
|  | Adjusted BIC | 21072.787 | 20162.590 | 20170.926 |
|  | Entropy | 0.690 | 0.943 | 0.922 |
|  | Negative LL | 10491.405 | 10027.308 | 10022.479 |
|  | LMR *P* value | .000 | .000 | .007 |
|  | Class counts | Class 1: 623 (28.9%) | Class 1: 742 (34.4%) | Class 1: 804 (37.3%) |
|  |  | Class 2: 1533 (71.1%) | Class 2: 802 (37.2%) | Class 2: 730 (33.9%) |
|  |  |  | Class 3: 612 (28.4%) | Class 3: 612 (28.4%) |
|  |  |  |  | Class 4: 10 (0.5%) |
| Note*.* AIC, Akaike information criterion; BIC, Bayesian information criterion; LL, log likelihood; LMR, Lo‐Mendell‐Rubin likelihood ratio test. | | | | |
